# The Potency of *Moringa oleifera* Lam. as Protective Agent in Cardiac Damage and Vascular Dysfunction

**DOI:** 10.3389/fphar.2021.724439

**Published:** 2022-01-24

**Authors:** Fenty Alia, Mirasari Putri, Neni Anggraeni, Mas Rizky A. A Syamsunarno

**Affiliations:** ^1^ Study Program of Biomedical Engineering, School of Electrical Engineering, Telkom University, Bandung, Indonesia; ^2^ Department of Biochemistry, Nutrition, and Biomolecular, Faculty of Medicine, Universitas Islam Bandung, Bandung, Indonesia; ^3^ Medical Laboratory Technologist, Bakti Asih School of Analyst, Bandung, Indonesia; ^4^ Department of Biomedical Sciences, Faculty of Medicine, Universitas Padjadjaran, Jatinangor, Indonesia

**Keywords:** antioxidant, anti-inflammation, anti-apoptosis, cardiac damage, *Moringa oleifera*, quercetin, vascular dysfunction, N,⍺-L-rhamnopyranosyl vincosamide

## Abstract

Cardiac damage and vascular dysfunction due to underlying diseases, such as hypertension and cardiac thrombosis, or side effects from certain drugs may lead to critical illness conditions and even death. The phytochemical compounds in natural products are being prospected to protect the heart and vascular system from further damage. *Moringa* genus is a subtropical tree native to Asia and Africa, which includes 13 species; *Moringa oleifera* Lam. (MO) is the most cultivated for its beneficial uses. MO is also known as the “miracle tree” because it has been used traditionally as a food source and medicine to treat various diseases such as anemia, diabetes, and infectious or cardiovascular diseases. The phytochemical compounds identified in MO with functional activities associated with cardiovascular diseases are N,α-L-rhamnopyranosyl vincosamide, isoquercetin, quercetin, quercetrin, and isothiocyanate. This study aims to investigate the potency of the phytochemical compounds in MO as a protective agent to cardiac damage and vascular dysfunction in the cardiovascular disease model. This is a scoping review by studying publications from the reputed database that assessed the functional activities of MO, which contribute to the improvement of cardiac and vascular dysfunctions. Studies show that the phytochemical compounds, for example, N,α-L-rhamnopyranosyl vincosamide and quercetin, have the molecular function of antioxidant, anti-inflammation, and anti-apoptosis. These lead to improving cardiac contractility and protecting cardiac structural integrity from damage. These compounds also act as natural vasorelaxants and endothelium protective agents. Most of the studies were conducted on *in vivo* studies; therefore, further studies should be applied in a clinical setting.

## 1 Introduction

Hypertension, cardiac thrombosis, and certain drugs can cause critical illness due to cardiac damage and dysfunction of the vascular system, leading to death (Çakmak and Demir, 2020). Cardiac and vascular disorders are classified as cardiovascular diseases (CVDs). As the underlying number one cause of death worldwide, 32% of the global population (17.9 million people) have died of CVDs, of which 85% were due to heart attacks and strokes (World Health Organization, 2021). The most risk factor for CVDs is overweight, defined by having body mass index (BMI) outside the normal range. On the other hand, being overweight can also cause many illnesses such as hypertension or high blood pressure that increase CVD risk (Tran et al., 2021).

Coronary heart disease is affected by the integrity and contractility of endothelial cell damage. The occurrence of endothelial dysfunction disrupts the mechanism of vascular homeostasis, resulting in blood vessels vasoconstriction, thrombosis, coagulation impairment, leukocyte adhesion, platelet activation, and oxidative stress formation that leads to inflammation. This condition triggers the accumulation of cholesterol plaque in the blood vessels known as atherosclerosis, one of the pathological processes of CVD (Favero et al., 2014; Thygesen et al., 2018).

Considering the mortality of CVD, reducing its development and progression is very important. Therefore, pharmacological treatments have been established (Mayr and Jilma, 2006; Lavie et al., 2018), as well as the study of natural products that contain various phytochemical compounds prospected to protect the heart and blood vessels from further damage (Shaito et al., 2020).

One such natural product is *Moringa oleifera* Lam. (MO). MO has been suggested as anti-apoptosis, improving cardiac contractility and protecting cardiac structural integrity from damage. In addition, MO could act as a cardioprotective by improving inflammation and oxidative stress (Aekthammarat et al., 2019; Khalil et al., 2020). This review aims to investigate the potency of the phytochemical compounds in MO as a protective agent to cardiac damage and vascular dysfunction in the CVD model.

## 2 *Moringa oleifera* Lam. at Glance

*Moringa oleifera* Lam. (MO) is one of the 13 species of *Moringa* genus that belongs to *Moringaceae* family (Swati et al., 2018). It is a subtropical tree native to Asia and Africa. It is mostly confined to the Sub-Himalayas but is now being cultivated worldwide attributed to its beneficial uses. It can grow fast in high-temperature regions and lands with a low water supply (Nouman et al., 2013, Nouman et al., 2014). The leaves, bark, roots, flowers, fruit, and seeds of this soft-wood tree have been utilized for its nutritional and medicinal values (Das, 2012). Since the first international conference of MO was held in 2001, this plant has been widely examined and labeled as “miracle tree,” “natural gift,” or “mother’s best friend” (Leone et al., 2015).

Over the last thousand years, MO has been recognized as highly beneficial for improving wellness. The leaves, flowers, seedpods, seeds, roots, bark, and gum are used as food sources, traditional treatment of various illnesses, and to improve health (Gopalakrishnan et al., 2016; Khalid Abbas et al., 2018). However, leaves and seeds have the most pharmaceutical potential and nutritional and medicinal benefits (Leone et al., 2015). The leaves are rich in protein, minerals, and antioxidant compounds. People usually consume them in the form of fresh leaves or process them into powder. The leaves are taken as vegetables, snacks, herbal tea, and spice. The leaves are used as complementary food for babies and lactating women to prevent malnutrition and anemia (Idohou-Dossou et al., 2011; Baiyeri and Akinnagbe, 2013). In terms of traditional medicine, people use MO leaves for the treatment of infectious diseases, fever, high blood pressure, high blood sugar, male impotence, and skin diseases (Baiyeri and Akinnagbe, 2013). MO leaves are used not only for human consumption but also for animal nutrition and plant fertilizer (Abd El-Hack et al., 2018). The seeds have a high protein content and have hence been used to increase protein intake. Oil, as the main component of the seeds, can be collected traditionally by boiling shelled seeds with water or by using different extraction methods. The oil is known as “Ben oil” or “Behen oil”. The oil is not widely used as edible oil for cooking; it is usually utilized as non-food applications, such as skincare ingredients, biodiesel, or other oil mixtures due to its oxidative stability (Leone et al., 2016; Nadeem and Imran, 2016; Leone et al., 2018). This plant is known as a superfood based on its nutritional properties. Various research have reported that MO contains seven times more vitamin C than oranges, 10 times more vitamin A than carrots, 17 times more calcium than milk, nine times more protein than yogurt, 15 times more potassium than bananas, and 25 times more iron than spinach (Rockwood et al., 2013; Gopalakrishnan et al., 2016). MO leave can be used to treat malnutrition due to the high protein and fiber content (Khalid Abbas et al., 2018). Studies showed that the pods are fibrous, with 46.78% fiber and 20.66% protein. Although amino acids are more concentrated in flowers (∼31%) compared to pods (∼30%), the contents of palmitic, linolenic, linoleic, and oleic acids are comparable in flowers and immature pods (Gopalakrishnan et al., 2016). MO seed oil contains 76% PUFA that consists of linoleic, linolenic, and oleic acids (Lalas and Tsaknis, 2002). This dietary component may have remediating effects on metabolic syndrome by controlling cholesterol, reducing plasma triglycerides, and preventing CVDs (Burghardt et al., 2010).

A complete analysis of the nutrients of MO is shown in Table 1.

**TABLE 1:** Nutrition composition of *Moringa oleifera* (value in 100 g of plant materials) (Olagbemide and Philip, 2014; Gopalakrishnan et al., 2016).

Principle	Fresh leaves	Dry leaves	Leaf powder	Seed	Pods
Calories (cal)	92	329	205	—	26
Protein (g)	6.7	29.4	27.1	35.97	2.5
Fat (g)	1.7	5.2	2.3	38.67	0.1
Carbohydrate (g)	12.5	41.2	38.2	8.67	3.7
Fiber (g)	0.9	12.5	19.2	2.87	4.8
Vitamins					
Vitamin B1 (mg)	0.06	2.02	2.64	0.05	0.05
Vitamin B2 (mg)	0.05	21.3	20.5	0.06	0.07
Vitamin B3 (mg)	0.8	7.6	8.2	0.2	0.2
Vitamin C (mg)	220	15.8	17.3	4.5	120
Vitamin E (mg)	448	10.8	113	751.67	-
Electrolytes					
Potassium (mg)	259	1,236	1,324	—	—
Minerals					
Calcium (mg)	440	2,185	2003	45	30
Iron (mg)	0.85	25.6	28.2	-	5.3
Magnesium (mg)	42	448	368	635	24
Copper (mg)	0.07	0.49	0.57	5.20	3.1
Sulphur (mg)	—	—	—	0.05	137

MO not only is rich in nutrients but also contains anti-nutrients like flavonoids, which act as antioxidants (Stevens et al., 2016; Tiloke et al., 2018). Phytochemical compounds from all parts of MO are mainly flavonoids, phenolic acids, tannins, saponin, alkaloids, glucosinolates, and glycosides (Valdez-Solana et al., 2015; Brilhante et al., 2017; Paikra et al., 2017; Borgonovo et al., 2020). Various compounds from different parts of MO tree, such as leaves seeds, pods, bark, flowers, root, and stem that have been investigated, are shown in Table 2. These bioactive compounds act synergistically in their therapeutic effects, such as reducing blood glucose levels, anticancer, antibacterial, antifungal, neuroprotective, cardioprotective, anti-inflammatory properties, and modulating the immune system (Madi et al., 2016; Tiloke et al., 2018). MO leaves are high in polyphenols and rich in simple sugars, rhamnose, and glucosinolates and isothiocyanates (El-Massry et al., 2013). Meanwhile, MO seeds contain flavonoids (El-Massry et al., 2013) and known contain components that have hepatoprotectant activity, namely, gastrodigenin rhamnopyranoside (1-[O-(4-hydroxymethylphenyl)]-α-L-rhamno-pyranoside, GR) (Li et al., 2019; Sun et al., 2019).

**TABLE 2:** Phytochemical compounds of different parts of *Moringa oleifera* (Brilhante et al., 2017; Paikra et al., 2017; Borgonovo et al., 2020).

Plant part	Phytochemical compounds
Leaves	Quercetin, Kaempferol, 4-[(alpha-L-rhamnosyloxy)benzyl]isothiocyanate (Moringin), Niazirin, Niazirinin, Benzylglucosinolate
Seeds	Quercetin, Kaempferol, Moringin, Niazimin, Niazirin, 4-[(alpha-L-rhamnopiranosyloxy)benzyl]glucosinolate, ß-sitosterol
Pods	Moringin, ß-sitosterol
Bark	4-[(alpha-L-rhamnopiranosyloxy)benzyl]glucosinolate
Flowers	Quercetin, Isoquercetin, Kaempferol
Root	Quercetin, Kaempferol, Moringin, Moringinine, 4-[(alpha-L-rhamnopiranosyloxy)benzyl]glucosinolate
Stem	Quercetin, Kaempferol, ß-sitosterol

## 3 Pharmacokinetic Studies and Toxicity of *Moringa oleifera* Lam.

Pharmacokinetic studies of MO have not been carried out, especially those related to the absorption and elimination of MO. However, studies of the GR were conducted by Li et al. (2019). The pharmacokinetic studies of GR showed that the time for GR to reach a maximal concentration (Cmax) orally was 10 and 5 min intravenous. GR as much as 10 mg/kg was distributed in the tissue in the range of 5–30 min and eliminated within 3 h. The distribution of GR was mostly found in the heart, kidneys, and spleen. However, GR levels in the kidneys will decrease faster than other organs, which is about 30 min. The liver, lungs, and brain receive less GR distribution than the kidneys, heart, and spleen (Li et al., 2019).

MO both in leaves and bark did not show toxicity signs using acute, sub-acute, and chronic toxicity test (Awodele et al., 2012; Reddy et al., 2013). However, several studies have shown that the different solvents of MO extract affect the test. On the basis of the sub-acute toxicity study of MO using Swiss Albino rats, it was shown that ethanol solvent was safer than aqueous (Kasolo et al., 2012). Sub-acute toxicity of MO leaves using an aqueous extract showed signs of mild organ toxicity, as seen based on an increase in WBC, chloride, potassium, calcium ions (Cl^−^, K^+^, and Ca^2+^) concentrations, an increase in the average ALP (alkaline phosphatase), ALT (alanine aminotransferase), AST (alanine aminotransferase), and total bilirubin (Kasolo et al., 2012). An increase in AST also occurred in the acute toxicity of an aqueous—methanol extract of MO leaves in female Wistar Albino rats with a dose of LD50 [2,000 mg/kg body weight (BW)] (Okumu et al., 2016). Histopathological changes were also found upon examination in the form of focal hepatocyte swelling and necrosis (Kasolo et al., 2012; Okumu et al., 2016) in the area around the central and hepatic veins and hepatic vein congestion (Okumu et al., 2016). Histopathological changes were also found in the kidney and heart tissue (Kasolo et al., 2012). Meanwhile, the acute toxicity of aqueous extract of MO leaves on male Wistar Albino rats administered orally did not show any sign of toxicity, only at doses of 3,200–6,400 mg/kg BW showed a decrease in locomotion and dullness (Awodele et al., 2012). Administration of high doses of MO extract, both leaf and seed extracts, can show changes in several parameters such as blood parameters (WBC) (Kasolo et al., 2012) and organ damage parameters such as an increase in ALT and AST (Ajibade et al., 2012; Kasolo et al., 2012; Okumu et al., 2016). On the other hand, MO leaves and seeds in low doses showed relatively safe effects (Ajibade et al., 2012; Okumu et al., 2016).

## 4 Role of MO in Cardiovascular Diseases

**FIGURE 1:**
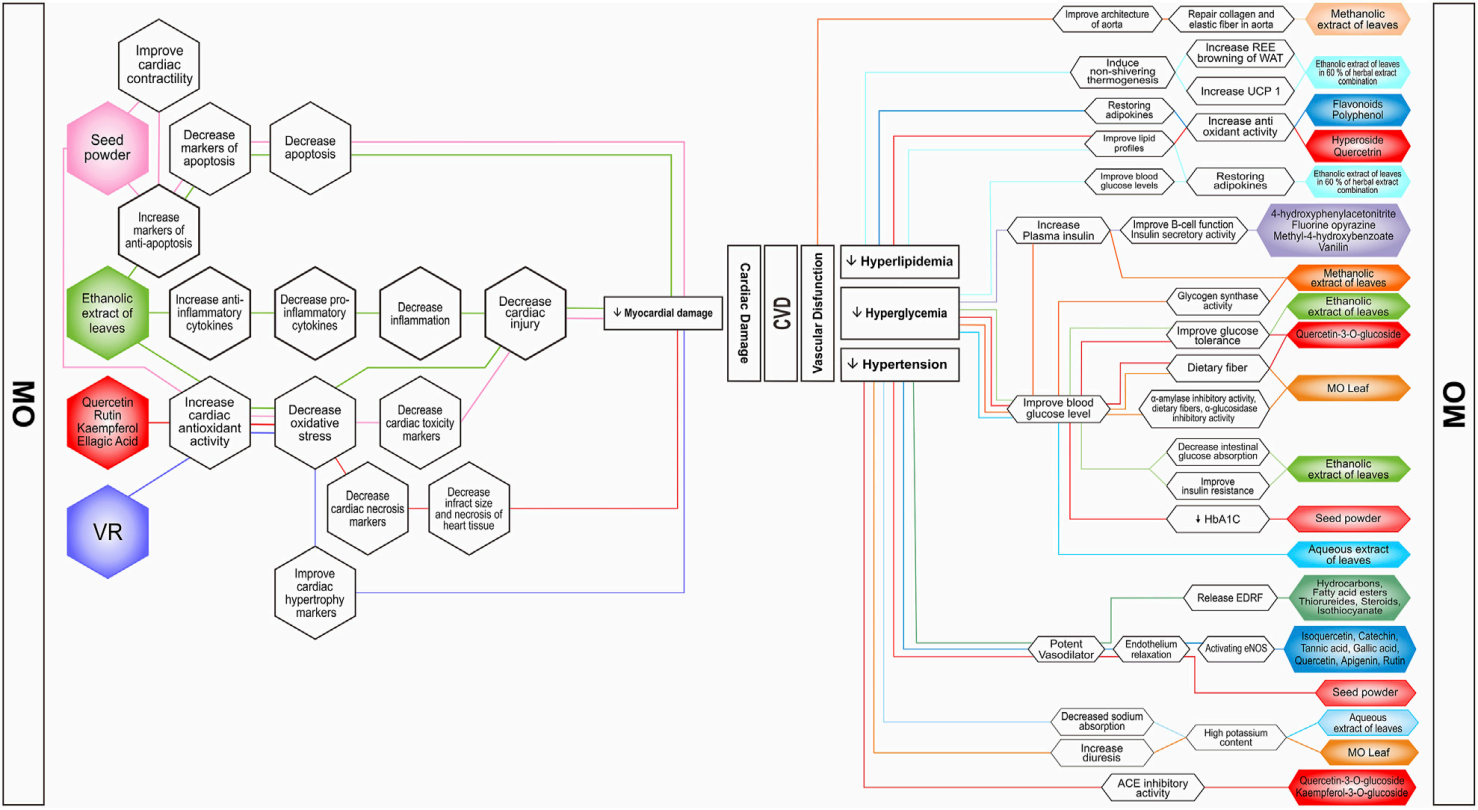
Possible mechanism of actions of *Moringa oleifera* nutrients and bioactive compounds in combating vascular dysfunctions and myocardial damages. The bioactive compounds prevent and improve CVDs’ risk factors such as hyperlipidemia, hyperglycemia, and hypertension. MO preparations are in the form of isolated metabolites, whole extracts, or pure parts. Notice that there is one promising approach to improve CVD risk by inducing non-shivering thermogenesis. ACE, angiotensin-converting enzyme; EDRF, endothelium-derived relaxing factor; eNOS, endothelial nitric oxide synthase; HbA1C, hemoglobin A1C; REE, resting energy expenditure; UCP1, uncoupling protein 1; VR, N,α-L-Rhamnopyranosyl vincosamide; WAT, white adipose tissue.

The occurrence of CVD is inseparable from the risk factors that influence, and this section describes the role of MO in CVD and the management of classic CVD risk factors, such as hypertension, obesity, hyperglycemia, and dyslipidemia, not only *in vitro* and *in vivo* but also in clinical settings. (Figure 1).

### 4.1 The Ability of MO to Treat Obesity and Obesity-Related Cardiometabolic Abnormalities

The management of CVD risk factors such as obesity, obesity-related cardiometabolic abnormalities, and hypertension has a role in minimizing CVD incidents (Aekthammarat et al., 2019). Obesity can trigger an oxidative stress condition due to an imbalance of pro-oxidants and antioxidants in the body. In obesity, there are excessive lipogenesis and inhibition of lipolysis. Fat, especially in the visceral area, has more glucocorticoid and androgen receptors, has increased metabolic activity, is sensitive to lipolysis, and is more prone to insulin resistance. This situation can lead to dyslipidemia, insulin resistance, and inflammation that cause endothelial dysfunction (Madkhali et al., 2019).

Several studies have demonstrated the antihyperlipidemic potential of MO. This lipid lowering effect can prevent atherosclerosis incidences. In animal models, two experiments showed remarkable improvement of BW and lipid profiles in the administration of MO leaves methanolic extract in doses of 200 and 400 mg/kg BW in rats. The first study showed that administration of the extract along with high-fat diet (HFD) feeding for 49 days led to a significant increase in HDL level and decrease of BW, total cholesterol, triglycerides, LDL level, and atherogenic index (Bais et al., 2014). This study found that alkaloids, tannins, flavonoids and terpenoids, and steroids contained in MO extract were responsible for the hypolipidemic effects, due to their antioxidant activities. However, further study needs to be performed for identification of specific compounds (Bais et al., 2014). The second study that used the same kind of extract in the same doses for 3 weeks after 9 weeks administration of HFD in rats showed the improvement in BW, BMI, Lee index, and HDL. There was also an improvement of hyperlipidemic parameters such as total cholesterol, triglyceride, LDL, and VLDL (Madkhali et al., 2019). Other important findings from this study were that administration in a dose of 400 mg/kg showed endothelium-mediated vasodilatation and improvement of aorta damage caused by HFD. MO decreased fat accumulation and repaired collagen elastic ratio, thus leading to almost normal architecture of the aorta (Madkhali et al., 2019). However, the last study did not perform phytochemical analysis to find the compounds that might be responsible for the lipid lowering activities. Both of the studies also used different extraction methods that might lead to obtaining different types and amounts of compounds (Nobossé et al., 2018). One *in vivo* study using MO ethanolic extract in a dose of 600 mg/kg BW orally for 12 weeks showed ameliorative effects in BW, atherogenic index, and insulin resistance in obese rats. This study provided molecular mechanisms to explain anti-obesity and anti-cardiometabolic abnormalities of polyphenolic and flavonoids antioxidant activities, by working directly on visceral mass and restoring mRNA expression of leptin, resistin, and adiponectin genes (Metwally et al., 2017). Cardiac ameliorative effect of MO in obese rats was investigated using administration of 200 and 400 mg of MO leaves methanolic extract orally for 12 weeks along with HFD. It was found that the extract decreased BW and improved TC, TG, HDL, and LDL profiles. In addition, the extract improved cardiac enzymes CK-MB, AST, and ALT, and decreased lipid peroxidase level by increasing SOD, CAT, and glutathione peroxidase (GPx) activities, possibly associated with antioxidant activity of phenolic compounds, mainly hyperoside and quercetrin contained in the extract (Mabrouki et al., 2020).

Research on obesity treatment has found new and promising targets. One of them is thermogenic adaptive tissue stimulation. Mitochondria in skeletal muscle and brown adipose tissue (BAT) plays a role in adaptive thermogenesis by generating heat during digestion and absorption of food to protect against environmental changes such as cold temperatures and is a thermal effect of exercise. Thereby, BAT stimulation and regulation are expected to reduce BW effectively (Tseng et al., 2010). Several natural compounds such as polyphenol and flavonoid derivatives can stimulate the proliferation and differentiation of BAT (Kang et al., 2018; Wood Dos Santos et al., 2018).

A study on MO has led to the stimulation of thermogenesis as a targeted therapy for obesity. A study using an herbal extract combination comprised of 60% MO leaves ethanolic extract in doses of 100 and 250 mg/kg BW for 28 days significantly reduced BW gain, total body fat mass, the fat cell size of inguinal and epididymal white adipose tissue (WAT), liver weight, and hepatic triglycerides and increased resting energy expenditure (REE) and fat oxidation on obese rats. The decrease of fat cell size and mass in supplemented obese rats was due to the increase energy metabolism through browning of WAT, the increase of expression of UCP1, and down-regulation expression of key adipogenic marker proteins (PPARγ, C/EBPα, CD36, aP-2α, and perilipin (Kundimi et al., 2020). However, they found that this herbal combination did not substantially affect the interscapular BAT fat mass of obese rats. This study has limitations; they did not explain whether this combined extract composition influences WAT browning or modulate BAT activation to increase energy metabolism. Another *in vivo* study demonstrated the effect of MO leaves ethanol extract on interscapular BAT. Mice were induced with HFD and given MO leaves ethanol extract in doses of 280 and 560 mg/kg BW for 7 weeks. It was found that BAT weight, BAT weight/BW ratio, BAT weight/WAT weight ratio, and BAT cell density were significantly increased in HFD-mice treated with MO compared to the HFD only mice. Also, the expression of Bone Morphogenetic Protein 7 (BMP7), a transforming growth factor–β (TGF-β) superfamily that can induce BAT adipogenesis and increase UCP1 expression, was measured. Mice given MO at dose 280 mg/kg BW significantly increase serum BMP7 levels. MO ethanol extract stimulated the development of BAT by upregulating serum BMP7 expression. This study suggested that flavonoids found in MO could modulate the BMP7 expression. However, this study did not provide phytochemical analysis, but they proposed that flavonoids were responsible for the mechanisms (Syamsunarno et al., 2021). These data were supported by an *in vitro* study that used human adipose-derived mesenchymal stem cells to investigate the effect of MO on lipids metabolism and antioxidant systems related to the adipogenesis process (Barbagallo et al., 2016). This study analyzed the expression of UCP1, Sirtuin 1 (SIRT1), peroxisome proliferator-activated receptor alpha (PPARα), and peroxisome proliferator-activated receptor-gamma coactivator 1 alpha (Pgc-1α) to evaluate the effect of MO on adipocyte thermogenic pathways. It was found that thermogenesis modulates improved metabolic rate, including glucose and lipid metabolism in humans. During adipogenic differentiation, the administration of MO significantly increased the mRNA expression of SIRT-1, PPARα, Pgc-1α, and UCP1 that indicated the activation of the heat-generating pathway, which is a lost proton pumping cycle through uncoupling proteins actions. They also measured the antioxidant activity of MO against DPPH. In particular, the percentage of DPPH inhibition resulted was about 81, 63, 42, 33, 13, and 18%, respectively, at a concentration of MO of 500–250-100–50-10–5 μg/ml. However, therapeutic efficacy of these antioxidant abilities must be confirmed by sufficient *in vivo* study due to the presence of several factors that can influence the antioxidant potential, such as physiopharmacological processes, that may lead to irrelevant results (Kasote et al., 2015). Finally, they concluded that MO has the potential to modulating lipid metabolism by upregulates the expression of SIRT-1, PPARα, Pgc-1α, and UCP1, the crucial proteins involved in thermogenesis (Barbagallo et al., 2016).

Some clinical studies were conducted to investigate the role of MO in metabolic abnormalities. Two randomized, double-blind placebo-controlled clinical studies in overweight/obese adults examined an interesting herbal extract combination comprised of 60% MO leaves ethanolic extract. The first study found that daily supplementation of this formulation in a dose of 900 mg for 8 weeks in 21 obese adults provided benefits in reducing BW and BMI, improved serum triglyceride concentration, LDL/HDL ratio, and fasting blood glucose levels. In addition, this study also found that 50 µg/ml of the extract exhibit anti-adipogenesis activity in 3T3-L1 mice adipocytes by upregulating adiponectin, Pentraxin-3, Pref-1, and down regulating MCP-1, resistin, and PAI-1 (Sengupta et al., 2012). The following study demonstrated the benefit of this herbal formulation in 66 healthy overweight adults. It was found that daily intake in a dose of 900 mg for 16 weeks also significantly reduced BW, BMI, total body fat, waist, and hip circumferences, LDL, and increased HDL level (Dixit et al., 2018). Those results above were possibly related to the presence of three significant compounds contained in the herbal extract combination, namely, quercetin-3-O-glucoside, mahanine, and curcumin. However, how these compounds act synergistically still needs to be explained, especially when considered safe for human consumption (Sengupta et al., 2012).

Obesity, hyperglycemia, and dyslipidemia are related to each other. Although hyperglycemia does not cause CVDs directly, the treatment of hyperglycemia should be considered prevention of CVDs. Several studies have been demonstrated the potential effect of MO in lowering blood glucose in some experiments using hyperglycemic animal models. A study presented the effect of MO leaf powder in reducing blood glucose levels. Goto-Kakizaki (GK) and Wistar rats were given glucose 2 mg/kg BW orally to assess oral glucose tolerance test (OGTT). Group 1 was only given glucose, and the other group was given glucose along with MO leaf powder at a dose of 200 mg/kg BW. The treatment group showed lower fasting blood glucose levels than GK rats without MO. Blood glucose levels significantly decreased at 20, 30, 45, and 60 min for MO-treated GK rats and 10, 30, and 45 min for MO-treated Wistar rats (*p* < 0.05) compared to both controls after glucose administration. Furthermore, they analyzed the types of polyphenols in MO leaf powder by HPLC with a photodiode array detector. The result showed a high concentration of quercetin-3-glycoside, rutin, kaempferol glycosides, and other polyphenols, likely chlorogenic acid. They suggested MO has improved glucose tolerance might be stimulated by quercetin-3-glucoside, and fiber contents in MO leave powder *via* inhibition of glucose uptake and slowing gastric emptying time (Ndong et al., 2007).

Similarly, a study used MO seed powder at doses of 50 and 100 mg/kg BW mixed with feed after 4 weeks of treatment decreased by 35% and 45% fasting blood glucose and 13% and 22% HBA1c compared to the control group, respectively. In addition, interleukin-6 (IL-6) and lipid peroxidation levels increased, whereas the activity of catalase, SOD, and GSH decreased in STZ diabetic rats. Nevertheless, in STZ, diabetic rats treated with MO almost recovered these parameters to normal levels. Although it was suggested that these therapeutic effects were due to the active ingredients present in Moringa seeds, such phenolic and flavonoids contained in MO seed powder have antioxidant activity; however, this study did not perform phytochemical analysis (Al-Malki and Rabey, 2015).

Administration of MO leaves aqueous extract at a dose of 200 mg/kg BW reduced fasting blood glucose and postprandial glucose levels by 26% and 30%, respectively, in OGTT compared to the untreated group. Another group, which gave MO at a dose of 300 mg/kg BW to diabetic rats for 21 days, decreased 69% of fasting glucose and 51% of postprandial glucose levels. In addition, elevated hemoglobin and total protein levels on long-term treatment with the extract for 21 days indicated a beneficial effect in reducing the severity of diabetes. Unfortunately, they did not elucidate the hypoglycemic effect and antidiabetic mechanism of the MO leaves aqueous extract (Jaiswal et al., 2009).

In addition to using aqueous extracts and powders from MO parts, several studies have also used methanol and ethanol extracts to investigate the effectiveness of MO in controlling blood glucose. A study using oral administration of ethanol extract of MO leaves (500 mg/kg) and glibenclamide (0.5 mg/kg) as a reference drug measured fasting blood glucose and glucose tolerance. The result showed that MO ethanol extract improved hyperglycemic and glucose tolerance at 30, 90, and 120 min after glucose (2.5 g/kg) solution gavage. Furthermore, they performed plasma insulin concentration, intestinal glucose absorption, and α-amylase activity of MO extract. The result showed that intestinal glucose absorption significantly reduced and no significant induction in insulin secretion. Although α-amylase activity assay showed no significant decrease in the starch’s catabolism, this promising result is important to perform with another proper method. Again, in this study, the mechanism of lowering the blood glucose effect of MO extract was not described (Azad et al., 2017). A significant decrease in blood glucose levels has been reported in treated STZ induced rats with MO leaves methanolic extract at a dose of 250 mg/kg BW for 6 weeks compared to diabetic control. However, they did not explain the mechanism of MO leaves methanolic extract in lowering blood glucose levels (Omodanisi et al., 2017).

Furthermore, another study demonstrated the effect of MO leaves methanol extract in diabetic rats. MO methanol extract at 300 and 600 mg/kg enhances glucose tolerance by 56 and 57%, and blood glucose levels were reduced by 76 and 64%, respectively, compared with the control diabetic group. Expectedly, in untreated diabetic rats, the insulin concentration was 46% lower compared with normal. In rats treated with MO at 300 and 600 mg/kg BW, plasma insulin increased by 1.6-fold (*p* < 0.01) and 1.7-fold (*p* < 0.01), respectively. Meanwhile, in untreated diabetic rats, the insulin concentration was 46% lower than normal rats, glycogen synthase activity in the liver and muscle tissues was significantly reduced, and glucose uptake was lower than normal. In rats treated with MO at doses of 300 and 600 mg/kg BW, plasma insulin increased by 1.6-fold (*p* < 0.01) and 1.7-fold (*p* < 0.01), respectively, glycogen synthase activities significantly improved in both the muscle and liver, and glucose uptake was inhibited by 49 and 59% respectively. In this study, they screened the phytochemical compounds of MO and measured the total phenolic and total flavonoids. These phytochemicals maintained beneficial effects such as reducing oxidative stress in diabetic rats, stimulating glucose transport, and inhibiting adipocyte differentiation (Olayaki et al., 2015).

Different part of MO has been evaluated as antidiabetic. The part that showed the most anti-hyperglycemic activity without causing side effects is the leaves. Natural compounds with antidiabetic activity such as phenolics, glucosinolates, isothiocyanate, syringic acid, gallic acid, and rutin are richly found in the leaves. Antidiabetic activity through insulin release activation has been reported from isolated compounds of MO extract. A study was conducted to evaluate the potential effect of pure compounds from parts of MO extract to improve insulin secretion in the islet pancreas of male BALB/C mice. The pure compounds that were isolated and characterized are 4-hydroxyphenylacetonitrite (roots), fluorine opyrazine, methyl-4-hydroxybenzoate (roots), and vanillin (stem barks). Under basal conditions (glucose 3 mM), insulin release was similar to islet control, and these compounds have not stimulated insulin production. However, when stimulated with glucose (16.7 mM), these pure compounds showed insulin secretory activity, but the highest was fluorine opyrazine, about 230%, whereas tolbutamide, a positive drug control, obtained 282.6% of insulin secretion. This study also demonstrated the ability of kaempferol to stimulate insulin secretion as a reference compound. The data showed that kaempferol significantly induced insulin secretion (377.2%) (Hafizur et al., 2018). This study also demonstrated the antihyperglycemic of these compounds *in vivo*. Rats induced with STZ (60 mg/kg i.v.) and nicotinamide (120 mg/kg i.v.) The diabetic rats were divided into six groups: the diabetic control group and the diabetic treatment group (2–5) were given compound suspension with a 25 mg/kg BW, respectively. The diabetic group was given glibenclamide (10 mg/kg) as a reference drug. After oral administration of the drugs, blood glucose concentration was measured at 0, 1, 2, and 3 h. There was no significant reduction in blood glucose concentration measured after the 3-h oral administration of methyl-4-hydroxybenzoate and vanillin to diabetic rats. On the other hand, there was a significantly decreased blood glucose concentration in doses and time-dependent manner after administration of 4-hydroxyphenylacetonitrite and fluorine opyrazine orally. Interestingly, fluorine opyrazine showed a more obvious effect on reducing blood glucose. Blood glucose decreased significantly starting at 2 and 3 h after gavage. Glibenclamide, the standard drug, lowered blood glucose more efficiently in a time-dependent manner. Furthermore, they evaluated fluorine opyrazine to improve β-cell function and elevate plasma insulin in type 2 diabetic rats. The data showed an increased insulin secretion occurred after 30 min of glucose administration (3 g/kg), when blood glucose is higher, but not in the absence of a glucose challenge. Fluorine opyrazine stimulated insulin secretion only at higher glucose concentrations and not at lower glucose concentrations. Furthermore, an index for β-cell function, the insulinogenic index (IGI), was measured. Fluorine opyrazine-treated rats (14.8 ± 1.55 pmol insulin/mmol glucose) were 3.8-fold higher than the control rats (3.9 ± 0.69 pmol insulin/mmol glucose). The IGI proposes that fluorine opyrazine treatment ameliorated β-cell function in diabetic rats (Hafizur et al., 2018). Although they performed the effect of pure compounds of MO to induce insulin secretion *in vivo* and *in vivo* study, they did not describe the isolation methods of the compounds.

Some human studies were performed to find the potency of MO to improve glucose metabolism. A preliminary study using 10 volunteers was performed to examine the effects of MO in insulin secretion in healthy subjects. Single-dose administration of 4-g MO leaf capsule orally after overnight fast significantly increased plasma insulin (Anthanont et al., 2016). However, the following randomized placebo-controlled study in therapy-naive of 32 patients with type 2 diabetes mellitus (T2DM) conducted by the same researchers did not demonstrate the blood glucose-lowering effects of MO. Administration of 8 g per day of MO leaf capsules for 4 weeks showed that MO leaf did not affect glycemic control (HbA1C level, fasting, premeal, and post-meal plasma glucose). The limitation of the study was the use of raw material without performing phytochemical analysis that might result in the lack of active substances responsible for blood glucose-lowering agents such as quercetin, chlorogenic acid, and moringinine. In addition, the dose used in this study was reported no adverse effects on the liver and kidney function of the subjects (Taweerutchana et al., 2017). Moreover, another study found that using raw material in administrating MO to the human body might lower blood glucose levels *via* another pathway. Administration of 20 g of MO leaf powder in traditional meals to 17 diabetic subjects showed an earlier peak of postprandial glucose response with lower increments at 90, 120, and 150 min and lower mean glycemic meal response than the non-diabetic group. One of the factors that attributed to these results is the ability of high total fibers contained in MO leaf to reduce the velocity of starch ingestion in the intestine, leading to a decrease of postprandial blood glucose, despite the ability of total polyphenols to inhibit α-amylase activity (Leone et al., 2018). For further study, there is a need to isolate the exact compounds responsible for the mechanism.

### 4.2 The Ability of MO to Lower Blood Pressure and Improve Endothelial Dysfunctions

Several studies were conducted to support the scientific evidence of the empirical use of MO to treat hypertension. Some studies examined the ability of MO as endothelial protecting agent using L-NAME (Nω-nitro-L-arginine-methyl ester) material as hypertension-inducer. L-NAME induces high blood pressure by acting as nitric oxide synthase (NOS) inhibitor (Aekthammarat et al., 2019; Aekthammarat et al., 2020). In a study using MO leaves aqueous extract orally in 30 and 60 mg/kg/day doses for 3 weeks in L-NAME-induced hypertension in rats showed a significant decrease in blood pressure and heart rate and also reduced the impairment of mesenteric arterial relaxation induced by acetylcholine. Rats that received 0.001–3 mg bolus injection of the extract showed dose-dependent vasorelaxation in the endothelium of mesenteric arterial beds. These emphasized the ability of MO as a potent vasodilator, probably by inducing the release of endothelium-derived relaxing factors (EDRFs). The extract also decreased vascular O_2_^−^ production and malondialdehyde (MDA) level in plasma and thoracic aorta and increased the level of SOD and CAT antioxidant enzymes, suggesting the potency of MO against oxidative stress-related hypertension in L-NAME rats (Aekthammarat et al., 2019). Further study was performed to investigate blood pressure-lowering activity of MO associated with enhanced NO production by delivering MO leaves aqueous extract in a 30 mg/kg intravenous dose after L-NAME administration in rats. MO showed longer period of BP-lowering effect possibly by activating eNOS *via* NOS-sGC dependent signaling. This condition increased NO availability, resulting in a vasorelaxing effect in the endothelium. This study provided quantitative analysis of some phenolic compounds. Although isoquercetin, catechin, tannic acid, gallic acid, quercetin, apigenin, and rutin were found using HPLC-DAD, further studies are needed to find specific compound which responsible for MO hypotensive activity (Aekthammarat et al., 2020).

Evidently, hypotensive activity of MO *via* releasing of EDRF also occurred under normal blood pressure condition. A study demonstrated the ability of MO root petroleum ether and dichloromethane extract to lower mean arterial pressure at a dose of 30 mg/kg when given intravenously in normotensive rats. Both extracts were suggested to cause the release of nitric oxide (NO) or EDRF in smooth muscle cells in different pathways. The petroleum extract stimulated muscarinic receptor and dichloromethane extract demonstrated to promote pathways other than cholinergic. GC-MS analysis showed the presence of hydrocarbons, fatty acid esters, thiorureides, steroids, and isothiocyanate in almost all extracts. These constituents may be responsible for hypotensive potential of MO root extracts (Sana et al., 2015).

Several human studies were conducted to investigate the blood pressure-lowering effect of MO. One human study was conducted to provide scientific evidence of MO blood pressure lowering effect. Healthy participants that were given 120 g of cooked MO leaves for a week showed a decrease of 2 h postprandial blood pressure compared to the control group. This study did not perform phytochemical analysis of MO leaves; however, the possible mechanisms of the blood pressure lowering effect may be attributed to MO free radical scavenging activity of nitrile, thiocarbamate, and isothiocyanate (Chan Sun et al., 2020). A clinic-based experimental study was performed to investigate the effect of MO aqueous extract in 30 normotensive adults. Consumption of MO aqueous extract (28.5, 57, and 85.7 mg/kg) orally on three different groups significantly lowered blood pressure and intraocular pressure at 30, 60, and 90 min after administration compared to baseline values, subsequently returned to baseline values after 150 min in a dose-dependent manner compared to control group that received distilled water only. This possibly occurred due to high potassium and calcium content in MO; therefore, it prevented the excessive absorption of sodium and decreased blood pressure (George et al., 2018). A study involving 20 males with stage-1 hypertension and obesity was performed to investigate the effect of 150 ml of MO leaf juice given twice a day for 30 days. It was found that MO leaf reduced systolic and diastolic blood pressure (DBP) significantly. This study did not perform phytochemical analysis; after there, they proposed that the effects were due to the ability of flavonoids to reduce oxidative stress, subsequently induced relaxation of resistant arteries (Sailesh et al., 2018). Another study showed that daily supplementation of 30 g of MO leaf powder in 30 obese hypertensive individuals for 60 days significantly decreased BMI, systolic blood pressure (SBP), and DBP and increased urine frequency by 27.2%. This study did not perform nutrient analysis and phytochemical analysis of the preparation; however, antioxidant activities generally attributed to bioactive compounds such as flavonoids, tannins, isothiocyanate, and thiocarbamate glycosides might be responsible for the decrease of BMI and blood pressure. It was noted that MO also demonstrated diuretic activity associated with its high potassium content (Fombang et al., 2016).

Another mechanism was proposed to explain the blood pressure-lowering effect of MO. By using *in vitro* ACE inhibition assay, MO was found to have ACE inhibitory activity attributable to two compounds contained in ethyl acetate leaves extract, namely, quercetin-3-O-glucoside and kaempferol-3-O-glucoside. This ACE inhibitory activity of these compounds was even higher than Captopril that served as positive control. Administration of this extract orally in 0.01 and 0.3 g/kg/day doses in L-NAME hypertensive rats showed a significant decrease of SBP after 4 weeks of treatment. This study also revealed that crude extract of MO is safe up to 2,000 mg/kg dosage (Acuram et al., 2019).

Endothelial dysfunction also develops during aging. A study revealed that administration of MO seed powder in a 750 mg/kg/day dose for 4 weeks gave protective effects to the endothelium function of aged rats. It was suggested that MO improved endothelium relaxation by increasing Akt signaling, activating endothelial NO synthase and down-regulating arginase-1 (Randriamboavonjy et al., 2019). Phytochemical analysis was not performed in this study, but the endothelium improvement effects were most likely due to polyphenol, glucosinolate, and isothiocyanate compounds contained in MO.

### 4.3 Ameliorative Effects of MO in Cardiac Toxicity Condition/Myocardial Infarction

Various studies have evaluated numerous natural products that might have cardioprotective effects by using different methods in animal models, such as surgery or drug-induced cardiotoxicity methods, to mimic human myocardial infarction (MI) (Panda et al., 2013; Panda, 2015). Application of drugs or chemical products such as anticancer, antibiotic, or pesticide also caused cardiotoxicity effects. These conditions encouraged researchers to investigate the role of natural compounds in combating MI by using the drugs or chemical compounds in animal model settings. Most of the studies concluded that the antioxidant activity of MO was responsible for its cardioprotective effects.

A study using specific alkaloid from MO leaves, N,α-L-rhamnopyranosyl vincosamide (VR), for the pretreatment of ISO-induced cardiotoxicity in rat models with a dose of 40 mg/kg BW orally for 7 days showed inhibition of the ST-segment elevation, normal heart rate, and decrease of necrotic cells of cardiac muscle (Panda et al., 2013). Administration of ISO caused elevation of MI biomarkers cTnT, CK-MB, LDH, and SGPT, indicating myocardial integrity damage. Furthermore, this condition could be countered by pretreatment of VR. This study also analyzed the free radical scavenging activities of VR *in vitro* and *in vivo*. VR decreased MDA and LOOH levels by increasing SOD, CAT, GPx, and GSH enzymes activities; that was the proposed mechanisms of cardioprotective effect of VR (Panda et al., 2013). Another study also investigated the free radical scavenging activities of VR. It was found that administration of magnetic hydrogel nanocomposite-loaded VR in doxorubicin (DOX)–induced cardiotoxicity rat models suppressed MDA by increasing GSH and SOD levels and decreasing mRNA level of cardiac hypertrophy marker ANP, BNP, and β-MHC mRNA in rat heart after 2 weeks intraperitoneal administration (Cheraghi et al., 2017). This justified the cardioprotective effect of VR mediated through its free radical scavenging activities.

Other studies using various doses of hydroalcoholic extract of MO leaves in MI animal models also showed the beneficial effects of MO in reducing myocardial damage, which was attributed to the antioxidant properties of MO. Pretreatment with butanolic fraction of MO leaves in a dose of 100 mg/kg/day for 28 days orally in ISO-induced cardiotoxicity rat models could lower MDA level and increase SOD, CAT, GPx, and total GSH. High concentration of quercetin, rutin, kaempferol, and ellagic acid contained in the extract was found responsible for these free radicals scavenging activities. These activities suppressed the increase of cardiac necrosis biomarkers LDH, CK-MB, and cTnT. This study also found the ability of the extract to reduce infarction, necrosis, and inflammation infiltrate in to the myocardium and to normalize myofibrillar structure through *in vitro* and *in vivo* analysis of cardiac tissue (Panda, 2015). A remarkable improvement of cardiac injury associated with antioxidant properties of ethanolic extract of MO leaves was also shown in benzene-induced leukemia rat models. This study aimed to assess the protective role of MO in cardiotoxic effects of DOX, an anticancer drug that is generally used to treat acute myeloid leukemia. Administration of 680 mg/day MO for 4 weeks orally in leukemia rat models significantly decreased MDA level and increased GSH and GPx levels. Interestingly, administration of MO in conjunction with DOX produced a higher ameliorative effect compared to the administration of MO alone. MO treatment significantly decreased cardiac toxicity markers ɤ-H2AX and ET-1 expressions in heart tissue, which could indicate a protective effect of MO against cardiac injury. A notably near to normal cardiac muscle appearance fibers with minute myolysis was realized from histological analysis. However, this study did not perform phytochemistry analysis to find specific MO compound, which may be responsible for the improvement of cardiac injury (Aniss et al., 2020). The seed, another interesting component of MO to explore, showed better survival rates and cardiac functions in mice with ligated left anterior descending branch of coronary artery, as a model for human MI (Li et al., 2020). Administration of MO seed powder in 600 and 900 mg/day oral doses for 28 days significantly alleviated contractile dysfunction by increasing the value of the left ventricular ejection fraction (LVEF) and left ventricular fractional shortening (LVFS). MO could also reduce infarct size and cardiac fibrosis; taken together, it might have occurred by antioxidative and antiapoptotic activities of MO compounds that were seen to decrease the apoptotic marker Bax, cytochrome-c, inducible NOS (iNOS) expression and NO level, and increase of Bcl2 marker (Li et al., 2020).

In addition to the ability to improve cardiac antioxidant status, MO has ability to ameliorate cardiac injury through its anti-inflammation and antiapoptotic potential. Administration of MO in cardiotoxicity rat models decreased inflammation markers TNF-α, NF-κB, and MCP-1 (Aniss et al., 2020). MI incident causes apoptosis and necrosis in cardiomyocyte that leads to histoarchitecture damage of cardiac tissue. MO compounds have been known to have antiapoptotic potential that may contribute to the prevention of cardiac tissue damage (Panda, 2015; Aniss et al., 2020; Khalil et al., 2020; Li et al., 2020). Administration of MO in cardiotoxicity rat models decreased apoptotic markers p53 and caspase 3 and increased antiapoptotic marker Bcl2 (Aniss et al., 2020).

**FIGURE 2:**
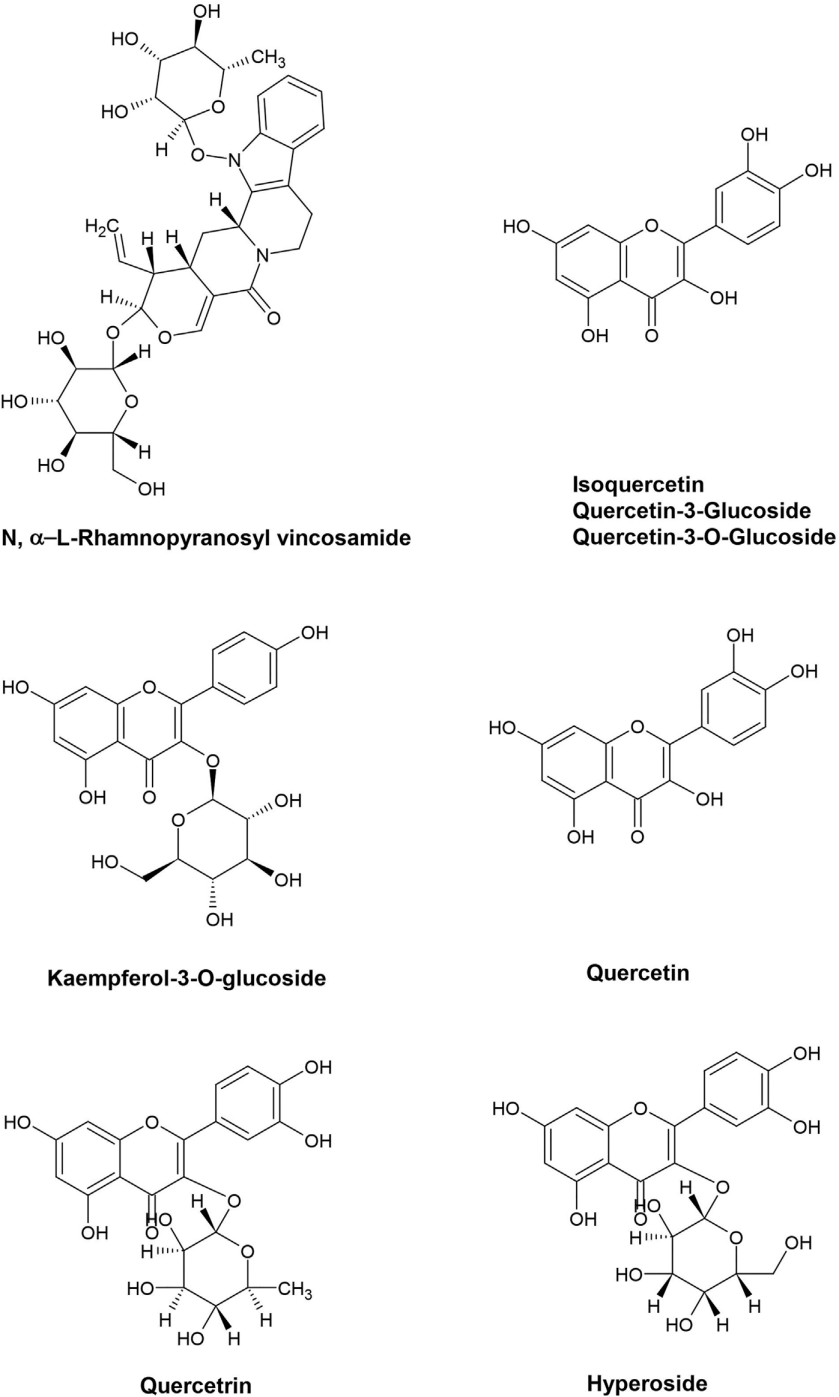
Chemical structures of prominent *Moringa oleifera* bioactive compounds that have role in combating CVDs.

The chemical structures of the prominent MO bioactive compounds described above can be seen in Figure 2. The studies evaluating the potency of MO for CVD are summarized in Tables 3, 4.

**TABLE 3:** Experimental approach of *Moringa oleifera* in CVD field.

No	MO parts	Bioactive compound	Dosage	Duration	RoA	References
1	Methanolic extract of leaves	Carbohydrates, alkaloids, tannins, saponins, flavonoids, triterpenoids, and steroids	200 and 400 mg/kg/day	49 days	Oral	Bais et al. (2014)
2	Methanolic extract of leaves	Not available	200 and 400 mg/kg/day	3 weeks (after HFD feeding)	Oral	Madkhali et al. (2019)
3	Ethanolic extract of leaves	Colorimetric assay: Polyphenolic (6.98 g of gallic acid equivalent/100 g), flavonoids (2.85 g of rutin equivalent/100 g)	600 mg/kg	12 weeks	Oral	Metwally et al. (2017)
4	Methanolic extract of leaves	HPLC-ESI-MS analysis: hyperoside (316,822 μg/g) and quercetrin (204,685 μg/g)	200 and 400 mg/kg BW	12 weeks	Oral	Mabrouki et al. (2020)
5	Ethanolic extract of leaves of in 60% of herbal extract combination comprised of MO, Murraya koenigii, Curcuma longa (Laila Nutraceuticals Ltd.)	HPLC analysis: 0.2% Quercetin-3-O-glucoside, 0.1% Mahanine, 0.7% Curcumin	100 and 250 mg/kg/day	28 days	Oral	Kundimi et al. (2020)
6	Ethanolic extract of leaves of in 60% of herbal extract combination comprised of MO, Murraya koenigii, Curcuma longa (Laila Nutraceuticals Ltd.)	HPLC analysis: 0.2% Quercetin-3-O-glucoside, 0.1% Mahanine, 0.7% Curcumin	50 µg/ml (*in vitro*)*	8 weeks (human study)*	Oral (human study)*#	*Sengupta et al. (2012)
			900 mg/day (human study)8#	16 weeks (human study)#		#Dixit et al. (2018)
7	Leave powder	HPLC analysis: quercetin-3-glycoside [1,494.2 µmol/100 g dry weight (dw)], rutin (1,446.6 µmol/100 g dw), kaempferol glycosides (394.4 µmol/100 g dw), and chlorogenic acid (134.5 µmol/100 g dw)	200 mg/kg	Single dose, examined 10, 20, 30, 45, 60, 90, and 120 min after administration	Oral	Ndong et al. (2007)
8	Seed powder	Not available	50 and 100 mg/kg	4 weeks	Oral	Al-Malki and Rabey (2015)
9	Aqueous extract of leaves	Not available	200 mg/kg (for OGTT model)	21 days	Oral	Jaiswal et al. (2009)
			300 mg/kg (for diabetic model)			
10	Ethanolic extract of leaves	Not available	500 mg/kg	Single dose, examined after 30, 90, and 120 min after glucose gavage	Oral	Azad et al. (2017)
11	Methanolic extract of leaves	Not available	250 mg/kg	6 weeks	Oral	Omodanisi et al. (2017)
12	Methanolic extract of leaves	Qualitative methods: saponin, flavonoids, steroids, phenol, glycosides, and tannins	300 and 600 mg/kg	6 weeks	Oral	Olayaki et al. (2015)
		Quantitative methods: total phenolic and total flavonoid				
13	Pure compounds from root and stem bark	4-hydroxyphenylacetonitrite (roots), fluorine opyrazine, methyl-4-hydroxybenzoate (roots), and vanillin (stem barks)	*In vitro*: 200 µm	Right after stimulated with glucose	Islet pankreas	Hafizur et al. (2018)
			*In vivo*: 25 mg/kg	Examine 0,1, 2, and 3 h after gavage	Oral	
14	Leaves	Not available	4 g	Single dose	Oral	Anthanont et al. (2016)
15	Leaves	Prosky method: total fiber (32.8 ± 0.5 g/100 g), soluble fiber (5.7 ± 0.1 g/100 g), and insoluble fiber (27.1 ± 0.2 g/100 g)	20 g	Single dose	Oral	Leone et al. (2018)
		UV-Vis spectrophotometry: total polyphenols (23.91 ± 0.2 mg GAE/g), total glucosinolates (21.22 ± 3.7 mg SE/g), and total saponins (16.92 ± 0.6 mg OAE/g)				
16	Aqueous extract of leaves	AAS model-solar 969 unicam series (acetylene flame): potassium (1,324 mg/100 g), calcium (2,003 mg/100 g)	28.5, 57, and 85.7 mg/kg	Single dose	Oral	George et al. (2018)
17	Leaf juice	Not available	150 ml	Twice daily for 30 days	Oral	Sailesh et al. (2018)
18	Leaf powder	Not available	30 g	60 days	Oral	Fombang et al. (2016)
19	Aqueous extract of leaves	HPLC-DAD-based assay: cathechin, gallic acid, isoquercetin, quercetin, tannic acid, and small amount of apigenin and rutin	MOE (30 and 60 mg/kg/day), Bolus injection of MOE (0.001–3 mg)	3 weeks	Oral, intra-arterial	Aekthammarat et al. (2019)
20	Aqueous extract of leaves	HPLC: (in mg/100 g dry extract): isoquercetin, 81.14; catechin, 76.77; tannic acid, 63.28; gallic acid, 21.23; quercetin, 20.47; apigenin, 4.03; and rutin, 0.11	Doses: 1, 3, 10, and 30 mg/kg of extract cumulative	30 min after administration of L-NAME	Intravenous	Aekthammarat et al. (2020)
21	Petroleum ether (MRP and MRP-1) and dichloromethane extracts (MRDC-IN, MRDC, and MRDC-1) of roots	GC-MS: isocyanate, isothiocyanate, thioureido, pyridine, and sesquiterpene	3 and 30 mg/kg	—	Intravenous with dose increment 60 and 120 s	Sana et al. (2015)
22	Cooked leaves	Not available	120 g of cooked *M. oleifera* leaves	A week	Oral	Chan Sun et al. (2020)
23	Crude methanol extract (ME), Ethyl acetate extracts (EA) of leaves	RP-HPLC: Quercetin-3-O-glucoside and Kaempferol-3-O-glucoside	ME (0.3 g/kg/day), ME (0.01 g/kg/), and EA (0.3 g/kg/day)	49 days: Three weeks after oral administration of L-NAME to mice, five of these animals were randomly selected and treated with ME or EA extracts (0.3 g/kg/day) during 25 days	Oral	Acuram et al. (2019)
24	Seed powder	Not available	750 mg/kg/day	4 weeks	Oral	Randriamboavonjy et al. (2019)
25	Leave extract	N,α-L-rhamnopyranosyl vincosamide (VR)	VR 40 mg/kg BW	7 days	Oral	Panda et al. (2013)
26	Leave extract	N,α-L-rhamnopyranosyl vincosamide (VR)—an indole alkaloid	200, 400, 800, 1,000, and 2,000 μg/ml	2 weeks	Intra-peritoneal	Cheraghi et al. (2017)
27	Butanolic fraction of leaves	HPLC analysis: Quercetin (980.16 μg/g), rutin (370 μg/g), kaempferol (490.5 μg/g), and ellagic acid 120.1 μg/g)	50, 100 and 150 mg/kg/day	28 days	Oral	Panda (2015)
28	Ethanolic extract of leaves	Not available	680 mg/day	4 weeks	Oral	Aniss et al. (2020)
29	Seed powder	Not available	600 mg/day or 900 mg/day	2 weeks prior to surgery and 4 weeks after surgery	Oral	Li et al. (2020)

**TABLE 4:** Functional activities of *Moringa oleifera* in CVD field.

No	Functional activities	Biological model	MO effects	References
1	Treatment of obesity and obesity-related cardiometabolic abnormalities	High-fat diet (HFD)–fed rat model	↓ Body weight (BW), total cholesterol (TC), triglycerides (TG), low density lipoprotein (LDL), and atherogenic index	Bais et al. (2014)
			↑ High density lipoprotein (HDL)	
		HFD-induced dyslipidemia rat model	↓ BW, Lee Index, BMI, TC, TG, LDL, and VLDL	Madkhali et al. (2019)
			↑ HDL endothelium-mediated vasodilatation improved architecture of aorta	
		Obese female rats	↓ BW, atherogenic index, insulin resistance, leptin, and resistin	Metwally et al. (2017)
			↑ Adiponectin	
		HFD-induced obesity rats	↓ BW, TC, TG, LDL, CK-MB, AST, ALT, and lipid peroxidation levels	Mabrouki et al. (2020)
			↑ HDL, SOD, CAT, and GPx activities	
		Obese rats	↓ BW, total body fat mass, WAT fat mass and cell size, liver weight, hepatic triglycerides, and leptin serum level	Kundimi et al. (2020)
			↑ Resting energy expenditure (REE), fatty oxidation	
			Browning of WAT	
			↑ UCP1	
			↓ PPARγ, C/EBPα, CD36, and ap-2α, perilipin	
		Overweight/obese adults; 3T3-L1 adipocyte	↓ BW, BMI, TG, and LDL/HDL ratio	Sengupta et al. (2012)
			↓ Fasting blood glucose	
			↑ Adiponectin, pref-1 protein	
			↓ Resistin, PAI-1	
		Overweight/obese adults	↓ BW, BMI, total body fat, waist and hip circumference, and LDL	Dixit et al. (2018)
			↑ HDL	
		GK and Wistar rats	↓ Fasting blood glucose	Ndong et al. (2007)
			Improve glucose tolerance	
		STZ-induced diabetic rats	↓ Fasting blood glucose and HbA1C	Al-Malki and Rabey (2015)
			↓ IL-6 and lipid peroxidase	
		OGTT and diabetic rat model	↓ Fasting blood glucose and post-prandial blood glucose	Jaiswal et al. (2009)
		Type 2 diabetic rats	↓ Blood glucose	Azad et al. (2017)
			Improve glucose tolerance	
			↓ Intestinal glucose absorption	
		STZ-induced diabetic rats	↓ Blood glucose	Omodanisi et al. (2017)
		Diabetic rats	↓ Blood glucose	Olayaki et al. (2015)
			Improve glucose tolerance	
			↑ Plasma insulin and glycogen synthase activity	
		Diabetic mice	Insulin secretory activity of islet pancreas	Hafizur et al. (2018)
			Improve β-cell function of the pancreas	
			↓ Blood glucose	
			↑ Plasma insulin	
		Healthy subjects	↑ Plasma insulin	Anthanont et al. (2016)
		Diabetic subjects	Earlier peak of post-prandial glucose response	Leone et al. (2018)
			Lower mean glycemic meal response	
			α-amylase inhibitory activity	
2	Natural vasodilator and improve endothelial dysfunctions	Nω-nitro-L-arginine-methyl ester (L-NAME)–induced high blood pressure in rats	↓BP, HR, vascular O_2_^−^ production, and MDA level in plasma and thoracic aorta	Aekthammarat et al. (2019)
			↑SOD and CAT dose-dependent vasorelaxation in endothelium of mesenteric arterial beds	
		Rats that administered with L-NAME *via* catheter	↓BP possibly by activating eNOS *via* NOS-sGC dependent signaling	Aekthammarat et al. (2020)
		Normotensive rats	↓ Mean arterial blood pressure (MABP)	Sana et al. (2015)
		Healthy human	↓2 h postprandial blood pressure	Chan Sun et al. (2020)
		Normotensive adults	↓ BP and intraocular BP	George et al. (2018)
		Stage-1 hypertension subjects	↓ Systolic blood pressure (SBP) and diastolic blood pressure (DBP)	Sailesh et al. (2018)
		Obese hypertensive individual	↓ BMI, SBP, and DBP	Fombang et al. (2016)
			↑ Urine frequency	
		Nω-nitro-L-arginine methyl ester (L-NAME)–induced high blood pressure in rats	↓ SBP	Acuram et al. (2019)
			ACE inhibitory activity	
			Improved endothelium relaxation	Randriamboavonjy et al. (2019)
		Middle-age Wistar rats and young Wistar rats	↑ Akt signaling and endothelial NO synthase	
			↓ Arginase-1	
3	Ameliorative effects in cardiac toxicity/cardiac infarction	Isoproterenol (ISO)–induced cardiac toxicity rat model	Inhibited ST-segment elevation and normalized HR	Panda et al. (2013)
			↓ Serum cTnT, CK-MB, LDH and SGPT, MDA, and LOOH	
			↑ SOD. CAT, GPx, and GSH	
		Doxorubicin-induced cardiac toxicity rats	↓ MDA level and mRNA level of cardiac hypertrophy markers (ANP, BNP, and β-MHC)	Cheraghi et al. (2017)
			↑ GSH and SOD	
		Isoproterenol (ISO)–induced cardiotoxicity rat model	↓ MDA, LDH, CK-MB, and cTnT	Panda (2015)
			↑ SOD, CAT, GPx, and total GSH	
		Benzene-induced leukemia rat model	↓ MDA	Aniss et al. (2020)
			↑ GSH and GPx	
			↓ TNF-α, NF-κB, and MCP-1	
			↓ p53 and caspase 3	
			↑ Bcl2	
			↓ cardiac ɤ-H2AX and ET-1	
		Myocardial infarction (MI) mouse model by surgery	Higher survival rate	Li et al. (2020)
			↑ LVEF and LVFS	
			↓ Infarct size and fibrotic scarring, apoptotic markers Bax, and cytochrome-c	
			↑ Bcl2	
			↓ Expression of iNOS	
			↓ The expression of gp91phox	
			↓ NO level	

## 5 Discussion

The potency of MO to overcome CVD is especially through its ability as free radical scavenger, anti-inflammation, and antiapototic agent, which is associated with the richness of bioactive compounds, such as flavonoids, phenolic acids, tannins, saponin, alkaloids, glucosinolate, and glycosides with their various isolates such as quercetin, N,α-L-rhamnopyranosyl vincosamide, and isothiocyanate (Figure 1). MO becomes a promising resource in finding natural therapy, even for MI, which is known as the main cause of death by CVD. According to The Fourth Universal Definition of In Myocardial Infarction, the pathological analysis denotes MI as myocardial cell death due to prolonged ischemia (Thygesen et al., 2018). MO increased cardiac antioxidant status, which led to the improvement of MI biomarkers, cardiac contractility, morphology of cardiac muscle, inflammatory cytokines, and apoptotic markers (Panda et al., 2013; Panda, 2015; Khalil et al., 2020; Saka et al., 2020).

Regardless of how protocols of research using natural ingredients are performed, it is still important to find specific compounds responsible for CVD-related mechanisms. The studies that described the MO potency in CVD concluded that quercetin, N,α-L-rhamnopyranosyl vincosamide, and isothiocyanate are the promising compounds for further study.

Quercetin, one of the typical active substances in MO, is a natural flavonoid found in many fruits and vegetables. Previous studies have shown the beneficial effect of quercetin in treating atherosclerosis events *via* reducing oxidative stress, lipid metabolism modulation, and as anti-inflammatory (Deng et al., 2020). In reducing oxidative stress, quercetin can directly act as an antioxidant by inhibiting activation of NADPH oxidase and p47phox expression (Xiao et al., 2017) and increase the production and activity of antioxidants, for example, as enzyme heme oxygenase-1 (HO-1) (Luo et al., 2020), vascular endothelial NO synthase (Loke et al., 2010), and glutamate–cysteine ligase (Li et al., 2016). In addition, quercetin attenuates the pro-oxidants, such as MDA and superoxide (Loke et al., 2010). Quercetin can also mitigate atherosclerotic inflammation through modulating galectin-3 (Gal-3)-pyrin domain-containing 3 (NLRP3) (Li et al., 2021), and 5′adenosine monophosphate-activated protein kinase (AMPK)/sirtuin 1 (SIRT1)/NF-κB signaling pathway (Zhang et al., 2020) inhibits lipid droplet formation by regulating proprotein convertase subtilisin/kexin type 9 (PCSK9), ATP-binding cassette sub-family G member 1 (ABCG1), and ATP-binding cassette transporter 1 (ABCAl) (Li et al., 2018). Furthermore, quercetin can ameliorate atherosclerotic lesions by altering the gut microbiota and depressing atherogenic lipid metabolites (Nie et al., 2019).

Alkaloid is a group of pharmacologically active compounds found in medicinal plants that mostly contain of basic nitrogen atoms (Vergara-Jimenez et al., 2017). Alkaloids successfully isolated from MO leaves are pyrrolemarumine 4″-O-α-L-rhamnopyranoside and 4′-hydroxyphenylethanamide (marumosides A and B) from pyrrole class (Sahakitpichan et al., 2011) and N,α-L-rhamnopyranosyl vincosamide from indole class (Panda et al., 2013). Alkaloids possess antioxidant, anticarcinogenic, antiviral, antifungal, and antimicrobial activities (Thawabteh et al., 2019) and also antiapoptotic and anti-inflammatory effects (Shrivastava et al., 2013). Examination using DPPH, ABTS^+^, chelating and reducing power assays has showed antioxidant activity of alkaloids (Liu et al., 2014). In a study using Parkinson’s rat models, alkaloid showed the ability to block the release of apoptogenic factors (cytochrome-c, caspase 3, and caspase-9), maintained the ratio of antiapoptotic factor Bcl2 with proapoptogenic factor Bax, and depleted proinflammatory cytokines TNF-α and IL-1ß, which were consistent with its antioxidant properties (Shrivastava et al., 2013). Alkaloids also showed vascular protection effects by significantly decreasing atherosclerotic lesion (Zhang et al., 2018) and caused endothelium relaxation *via* the activation of the potassium channels and reduction of calcium influx (Romero et al., 2019). Total alkaloids of MO leaves showed antihypertensive effect possibly *via* calcium channel blocker activity in isolated frog heart and tenia coli of guinea pig; however, this study did not confirm the specific alkaloid, which was responsible for the activity (Dangi et al., 2002). To the best of our knowledge, N,α-L-rhamnopyranosyl vincosamide is the first MO specific alkaloid that has been examined for its ability to counter cardiac damage. Further study is needed to explore any MO specific alkaloids for their CVD-related activity.

The underlying pathophysiology of CVD such as obesity, hypertension, and atherosclerosis occurred because of inflammation, with its wide array of cytokines secreted by immune cells (Tran et al., 2021). MO leaves extract could modulate humoral and cellular immunity in rats and mice (Gupta et al., 2010; Sudha et al., 2010). One of the interesting phytochemical compounds in MO leaves extract is isothiocyanates. Isothiocyanates formed by enzymatic hydrolysis of the MO glucosinolate (Mbikay, 2012; Waterman et al., 2014), and it has been shown to have strong anti-inflammatory properties. A study conducted using RAW 264.7 macrophage cell model of LPS-induced inflammation showed that MO isothiocyanates reduced iNOS and IL-1ß mRNA expression and inhibited the production of NO (Mbikay, 2012). Similarly, a reduction in gene expression of *iNOS*, *IL-1β*, and *IL-6* without affecting cell viability was reported (Jaja-Chimedza et al., 2017). An *in vitro* study reported a dose-dependent reduction in iNOS protein expression following incubation for 18 h of RAW 264.7 lipopolysaccharide-activated cells treated with different isothiocyanate concentrations from MO fruits (Cheenpracha et al., 2010). The MO anti-inflammatory activity was also examined using RAW 264.7 mouse macrophage cells, and it showed a reduction in iNOS expression, as well as a decrease in cyclooxygenase-2 (COX-2) (Park et al., 2011). These reported studies demonstrated that MO has potential in treating inflammatory-related diseases, including CVD.

## 6 Limitations and Recommendation for Future Research

All the studies mentioned above are conducted in different sample preparation, such as isolated metabolites, whole extracts, and pure MO parts, and have notable different dose range tested. There are also significant variations in the nutritional value and phytochemical content of MO. They depend on the genetic background, location, climate, and environmental factors (Valdez-Solana et al., 2015; Sultana, 2020). Furthermore, it is necessary to choose the extraction method and the solvent use because it will produce different quantity of bioactive compounds and produce different free radical abilities. For example, a study conducted on MO leaves revealed that extracts with ethanol and methanol solvents produced higher antioxidant activity than using water extract (Nobossé et al., 2018). Therefore, extraction technique must also be chosen to find suitable extract with high antioxidant activity. The identification of the plant and the use of full botanical taxonomic name are also important, especially in experimental study, to avoid inconsistency between studies.

MO is traditionally consumed in raw materials, such as fresh leaves or leaves powder, manufactured commercially by the community (Syamsunarno et al., 2021). These preparations are also applied in various human studies and become a challenge in implementing studies that use oral administration methods due to the unpleasant odor and intolerable taste of MO leaves. The acceptability test of the MO preparation and choosing aqueous extract (“tea”) or other odor and flavor masking methods are needed due to the subjects’ convenience and relate to dose determination (Leone et al., 2018; Fahey et al., 2019), without omitting the benefits of the compounds. The use of MO alcoholic extracts is still in the scope of *in vitro* or *in vivo* studies, so the utilization of these extracts in human studies must undergo strict supervision due to possible undesired effects.

Nanotechnology drug delivery becomes a new promising formulation in CVD studies due to better pharmacokinetic profiles, biocompatible, low toxicity and antigenicity, and more efficiency to target mitochondria dysfunctions (Forini et al., 2020). However, this technology is still limited to preclinical studies. Surprisingly, this drug-carrier technology application was found in some MO studies. Application of magnetic hydrogel nanocomposite for delivering MO bioactive compound showed a higher pharmacokinetic profile than that of the extract alone (Cheraghi et al., 2017). An *in vitro* study that examined the cytotoxicity effect of MO against colon cancer found that incorporation of MO leaves extract with silver nanoparticles showed higher antioxidant activity compared to that of before the incorporation. This is possibly related to the ability of the preparation to increase total polyphenolic compounds (Shousha et al., 2019). Another previous study found that nano-formulations in MO seed oil have gained a higher cytotoxicity effect on various cancer cell lines through mitochondrial-mediated apoptosis with minimal harmful effects compared to that of the free seed oil (Abd-Rabou et al., 2016). Thus, it is highly expected that there will be MO preparation using nano formulations applied in human studies related to CVD management purposes.

Other limitation is not all studies performed toxicity analysis of MO extract/compound they used (Cheraghi et al., 2017; Aekthammarat et al., 2020). One review article analyzed the MO leaves’ efficacy and safety. MO leaves including the extracts has high rank of safety at the doses utilized based on various *in vitro*, *in vivo*, and human studies (Stohs and Hartman, 2015). Nevertheless, preliminary studies that find the right non-toxic dosage is important, whether *in vitro* or *in vivo* as screening for study feasibility, but still, further human study is required to determine the dose safety and efficacy (Stohs and Hartman, 2015).

Apart of the ability of MO bioactive compounds to combat CVDs, MO seed oil also has potency to be investigated for further study due to its high PUFA. MO oil contains approximately 5.6–6.2% behenic acid (Nadeem and Imran, 2016). A previous study showed that daily fat supplement consisted of behenic acid from behenate oil for 1 week tended to raise total cholesterol and triacylglycerol plasma of seven mildly hypercholesterolemic men compared to other vegetable oils (Cater and Denke, 2001). This fact led to inconsideration of behenic acid for treating CVD’s risk factors. Contrary, more recent findings from a cohort study involving 2,680 older adults showed that a higher level of circulating plasma phospholipid very long saturated fatty acid (VLSFA) behenic acid was associated with a 15% significant reduction in aging-related CVD events, namely, MI, heart failure, stroke, transient ischemic attack, and claudication possibly by lowering endogenous levels of shorter-chain ceramides (Bockus et al., 2021).

The traditional application of MO, such as blood pressure and glucose-lowering agents, which were mentioned earlier, has been proven through many studies. However, *in vitro* findings must be confirmed by sufficient *in vivo* and human studies, especially in clinical settings. In conclusion, there are several mechanisms of MO associated with CVD. MO has the ability to improve cardiometabolic abnormalities, hypertension condition, and endothelial dysfunctions, and, in animal model, it has a beneficial effect in cardiotoxicity condition/MI. Further studies are still needed to complete the limitations found in previous studies.
